# Prediction of Clinically Significant Cancer Using Radiomics Features of Pre-Biopsy of Multiparametric MRI in Men Suspected of Prostate Cancer

**DOI:** 10.3390/cancers13246199

**Published:** 2021-12-09

**Authors:** Chidozie N. Ogbonnaya, Xinyu Zhang, Basim S. O. Alsaedi, Norman Pratt, Yilong Zhang, Lisa Johnston, Ghulam Nabi

**Affiliations:** 1 Division of Imaging Science and Technology, University of Dundee, Dundee DD1 4HN, UK; 2 College of Basic Medical and Health Sciences, Abia State University, Uturu 441103, Nigeria; 3 Division of Population Health and Genomics, School of Medicine, University of Dundee, Dundee DD1 4HN, UK; 110023382xyz@gmail.com; 4 Statistics Department, University of Tabuk, Tabuk 47512, Saudi Arabia; Balsaedi@ut.edu.sa; 5 Cytogenetic Unit, Human Genetics Unite, Ninewells Hospital and Medical School, Dundee DD1 9SY, UK; norman.pratt@nhs.scot; 6 School of Science and Engineering, University of Dundee, Dundee DD1 4HN, UK; y.y.zhang@dundee.ac.uk; 7 Pathology Unit, Ninewells Hospital and Medical School, Dundee DD1 4HN, UK; lisa.johnston3@nhs.scot

**Keywords:** prostate, cancer, mpMRI, PIRADS, radiomics, Gleason score, imaging, biomarkers

## Abstract

**Simple Summary:**

Radiomics is the field of computer-based medical image analysis that incorporates various radiological imaging features, such as texture and shape parameters, from scans to derive algorithms. These mathematical algorithms have the potential to predict the biological characteristics of disease. In this study, we obtained quantitative imaging texture features of pre-biopsy multiparametric MRI of men suspected of prostate cancer and extracted from the T2WI and ADC images focusing on gray-level co-occurrence matrices (GLCM). These were correlated with the Gleason score of the histopathology of radical prostatectomy specimen, including the prediction of clinically significant prostate cancer. The knowledge gained through this prospective protocol-based study should facilitate establishing that GLCM texture features alone can be used as a biomarker for predicting the presence of clinically significant PCa.

**Abstract:**

Background: Texture features based on the spatial relationship of pixels, known as the gray-level co-occurrence matrix (GLCM), may play an important role in providing the accurate classification of suspected prostate cancer. The purpose of this study was to use quantitative imaging parameters of pre-biopsy multiparametric magnetic resonance imaging (mpMRI) for the prediction of clinically significant prostate cancer. Methods: This was a prospective study, recruiting 200 men suspected of having prostate cancer. Participants were imaged using a protocol-based 3T MRI in the pre-biopsy setting. Radiomics parameters were extracted from the T2WI and ADC texture features of the gray-level co-occurrence matrix were delineated from the region of interest. Radical prostatectomy histopathology was used as a reference standard. A Kruskal–Wallis test was applied first to identify the significant radiomic features between the three groups of Gleason scores (i.e., G1, G2 and G3). Subsequently, the Holm–Bonferroni method was applied to correct and control the probability of false rejections. We compared the probability of correctly predicting significant prostate cancer between the explanatory GLCM radiomic features, PIRADS and PSAD, using the area under the receiver operation characteristic curves. Results: We identified the significant difference in radiomic features between the three groups of Gleason scores. In total, 12 features out of 22 radiomics features correlated with the Gleason groups. Our model demonstrated excellent discriminative ability (C-statistic = 0.901, 95%CI 0.859–0.943). When comparing the probability of correctly predicting significant prostate cancer between explanatory GLCM radiomic features (Sum Variance T2WI, Sum Entropy T2WI, Difference Variance T2WI, Entropy ADC and Difference Variance ADC), PSAD and PIRADS via area under the ROC curve, radiomic features were 35.0% and 34.4% more successful than PIRADS and PSAD, respectively, in correctly predicting significant prostate cancer in our patients (*p* < 0.001). The Sum Entropy T2WI score had the greatest impact followed by the Sum Variance T2WI. Conclusion: Quantitative GLCM texture analyses of pre-biopsy MRI has the potential to be used as a non-invasive imaging technique to predict clinically significant cancer in men suspected of having prostate cancer.

## 1. Introduction

Prostate cancer (PCa) is the most common non-cutaneous cancer in men and the second most common global cause of cancer-related deaths in men, accounting for 7.1% of all cancer-specific deaths, as reported in 2018 [1]. Approximately 1,111,700 new cases and 307,700 PCa-specific deaths have been recorded annually [2], making PCa an important public health issue.

Prostate-specific antigen (PSA), the most commonly used biomarker, is not reliable for the detection and risk stratification of prostate cancer, because numerous prostate conditions, such as benign prostate hyperplasia (BPH), prostatitis and urinary tract infection, can cause a rise in PSA levels [3,4,5]. A raised PSA level, suggesting the possibility of prostate cancer, leads to a transrectal ultrasound-guided biopsy of the prostate gland to obtain samples for histopathological confirmation of diagnosis. The histopathological grading of PCa is based on cell appearance or tissue structural abnormalities viewed under a microscope, and the Gleason score (GS) grading system is then used to evaluate the organizational features and prognosis of the prostatic glands. Although GS has contributed in the diagnosis, management and prognosis of PCa, its accuracy from biopsy is only about 58.3% [6]. Moreover, it is affected by inter-and intra-observer variations, resulting in the whole process being less than ideal for the detection and risk stratification of PCa [7]. There are other challenges such as reporting issues, sampling errors and poor clinical interpretations [8]. Obtaining the Gleason score requires an invasive procedure [9]. Thus, there is an urgent need for a non-invasive test for classifying PCa grades, which in combination with histopathology, improves risk stratification and precision care for patients [10].

In recent years, multiparametric magnetic resonance imaging (MRI) has become a promising non-invasive imaging modality for PCa detection and characterization [11,12], using a grading system known as PIRADS scores to achieve this. The score ranges from one (very low probability of cancer) to five (very high probability of cancer). PIRADS have been reviewed and revised to PIRADS 2.0 by the American College of Radiology (ACR) and the European Society of Uro-Radiologists (ESUR), and was published in early 2015 [13]. While PIRADS 2.0 provides substantial information on the acquisition, interpretation and reporting of mpMRI of prostate, it does not eliminate the possibility of inter-reader variability, a known challenge with the previous version. Therefore, there is a need to improve lesion characterization in the future using a quantitative parameters-based radiomics approach [14]. One of the crucial steps in radiomics is the acquisition of prospective protocol-based good-quality images. While radiomics analyses studies using mpMRI have been reported, with most of them being retrospective using biopsied tissues as a reference standard [15,16,17,18], in contrast, ours is a prospective study with radical prostatectomy (RP) histopathology as a reference standard. We focused on gray-level co-occurrence features as most previous studies have demonstrated the importance of the texture features based on GLCM of MRIs (i.e., T2WI) as an indicator for the pathological differences in PCa [16,19].

The aims of the study were to investigate the role of GLCM texture features, derived from pre-biopsy mpMRI, in the prediction of clinically significant PCa.

## 2. Materials and Method

### 2.1. Target Population

This was a prospective study between November 2018 and December 2019. In total, 200 men were recruited with the following inclusion criteria: age of 40–75 at referral; with at least 10 years life expectancy; clinically localized PCa: PSA ≤ 20 ng/mL and/or abnormal DRE but <T3 disease; and the ability to provide informed consent.

The exclusion criteria were: unable to give informed consent; prior prostatic biopsy within 12 months; contraindications to biopsy; poor general health and life expectancy < 10 years, including previous diagnosis of acute prostatitis within 12 months; history of PCa; prior transurethral prostatectomy; contraindications to MRI (cardiac pacemakers, allergic reaction to gadolinium-based contrast, renal function with a baseline eGRF 30 mL/min, intracranial clips and claustrophobia); and previous hip replacement.

The outcome of the study was firstly to identify radiomic features that correlated with the Gleason score. Secondly, independent radiomic features that were associated with the presence of clinically significant prostate cancer. Clinically significant prostate cancer was defined as the presence of prostate cancer with the Gleason score ≥ 4 + 3 [20]. In addition, the predicted probabilities using radiomic features and Prostate Imaging-Reporting and Data System (PIRADS) in predicting significant prostate cancer were compared.

### 2.2. MultiParametric MRI (mpMRI) Image Acquisition

For this study, Institutional Caldicott approval (IGTCAL number 5816) was obtained and all experiments, including the study protocol, followed approved institutional guidelines. All men had imaging data with corresponding histopathology of radical prostatectomy (RP). The histopathology of RP specimens was reported by an experienced uro-pathologist. The mpMRI scan was acquired using a 3T scanner (TIM Trio, Siemens, Erlangen, Germany), while sequences included T2WI and DWI. The T2WI acquisition was conducted using a turbo-spin echo sequence with a resolution of about 0.5 mm in the plane with a slice thickness of 3.6 mm. The DWI was a single-shot echo planar imaging sequence with a resolution of 2 mm in-plane and 3.6 mm slice thickness with diffusion encoding gradients ×3 direction. However, an apparent diffusion coefficient (ADC) map was computed from DWI data (b values = 0, 100, 400, and 800 s/mm^2^). The PIRADS v2 score on mpMRI was recorded by an experienced uro-radiologist and was blinded to all patient’s pathology reports. The PIRADS v2.0 were classified as follows: clinically significant cancer highly unlikely to be present (score 1); clinically significant cancer unlikely to be present (score 2); clinically significant cancer equivocal (score 3); clinically significant cancer likely to be present (score 4); clinically significant cancer highly likely to be present (score 5) [21]. The radiologist was blinded to all patients’ clinic-pathological information. The mpMRI including T2WI, DWI with a corresponding ADC map and the dynamic contrast-enhanced (DCE) of the largest tumor of each patient was scored on a scale of 1–5 using PI-RADS v2.0. The DCE sequencing involved 3D fast gradient-echo sequences with temporal resolution of 4 s, using intravenous 2 mL/kg of Dotarem, a gadolinium-based contrast agent. The prostate images were aligned along the longest axis to match the histologic sectioning of the prostate gland following radical surgery.

### 2.3. Radiomic Feature Analysis

Each image was converted to DICOM format before importing this to the MATLABR2020b software (https://www.mathworks.com/downloads/web_downloads/ (accessed on 15 November 2018)). Texture features were extracted at a resolution of 320 × 320 × 19 voxels and the intensities within each ROI were normalized to a (0–1) range. Normalization was applied to allow all the data to appear on the same scale across all the ROI. Data were normalized between a 0 and 1 range by subtracting it from the minimum value of the dataset and dividing the difference of the maximum and minimum values of the dataset.

### 2.4. Segmentation

For consistency between the region of interest (ROI) in both the T2WI and ADC images, all depicted ROI were carefully manually delineated with the same criteria and visually validated by an expert radiologist with 10 years of experience in uro-radiology before the quantitative imaging features were extracted. The anatomical landmarks of the urethra, the ejaculatory ducts, the prostatic capsule and the well-delineated hyperplastic nodules were used as a reference for visual co-registration, and the ROI were drawn on the T2WI and ADC maps in a way to match the location of tumors on pathology maps.

### 2.5. Feature Extraction and Selection

Feature extraction and selection were performed using the MATLAB R2020b software. The derived T2WI and ADC texture features were from GLCM, a second-order statistic characterizing the spatial relationship between the intensity values within ROIs. In total, 22 quantitative imaging features were extracted from the computed GLCM of ROI. The GLCMs textural features were computed from each directional matrix, and the mean of each feature across the slices were derived. Lastly, the average of each feature across the four directions was calculated to remove possible differences in directionality. The ADC maps were calculated from the nonzero b-value DWI datasets (100, 400, and 800 s/mm^2^). To remove possible perfusion effects, the b-values = 0 s/mm^2^ image was excluded from the ADC map computation.

### 2.6. Histological Gleason Score

The GS were obtained from the radical prostatectomy (RP) specimen by an experienced pathologist. The radical prostate specimens for histology were sliced into patient-specific molds (3.6 mm axial slices), and hematoxylin and eosin staining of microsections was carried out to help correlate the adjustment between imaging and histology. The molds were fabricated using a 3D printer, as described in previous studies [22,23]. Each patient’s corresponding tumor lesion was given a pathology Gleason grade score rating, consisting of five groups, as defined previously by Esptein JI et al. [24]. The patient’s Gleason grade scores were subsequently reclassified into three groups [24,25].

### 2.7. Statistical Analysis

The patient’s age (in years), the prostate specific antigen (PSA) and the PSA density (PSAD) were collected. The radiomic features of the PIRADS and the gray-level co-occurrence matrix (GLCM) were measured using mp-MRI images. Radiomic features included 22 variables, which were: Angular Second Moment T2WI; Contrast T2WI; Correlation T2WI; Sum Square Variance T2WI; Inverse Difference T2WI; Sum Average T2WI; Sum Variance T2WI; Sum Entropy T2WI; Entropy T2WI; Difference Variance T2WI; Difference Entropy T2WI; Angular Second Moment ADC; Contrast ADC; Correlation ADC; Sum Square Variance ADC; Inverse Difference ADC; Sum Average ADC; Sum Variance ADC; Sum Entropy ADC; Entropy ADC; Difference Variance ADC; and Different Entropy ADC (Supplementary Materials).

A Kruskal–Wallis test was applied first to identify the significant radiomics features between the three groups of GS (i.e., G1, G2 and G3). Subsequently, the Holm–Bonferroni method was applied to correct and control the probability of false rejections. We then used Spearman’s rank correlation for each of the radiomic features and the GS groups. The values of the correlation were mostly between ±0.5 and ±0.5, indicating moderate correlation.

After significant GS correlated radiomic features were identified, a two-step logistic regression was performed to explore explanatory radiomic features of significant prostate cancer. First, GS correlated T2WI and ADC radiomic features from the Kruskal–Wallis test and the Holm–Bonferroni adjustment, and the PSAD and PI-RADS were individually put into a univariate logistic regression model where the outcome was defined as having significant prostate cancer or not. Statistically significant variables were then put into the multivariable logistic regression model. Odds ratio (OR), 95% confidence interval (95% CI) of odds ratio, and *p* value were recorded. The discriminative ability of the predictive model was tested by the receiver operating characteristics (ROC) curve and the concordance statistic (c-statistic) was presented. The C-statistic using significant radiomic features, PSAD and PIRADS in predicting significant prostate cancer were compared. A nomogram was constructed based on the statistically significant variables in the final model. The predicted probabilities of significant prostate cancer were plotted against observed probabilities to test the calibration of the model. A decision curve analysis and internal validation were applied to determine the benefit of the nomogram. Statistical analyses were conducted by SPSS V25.0 and R v4.0.4. The alpha level was set at 0.05 to determine two-tailed significance.

## 3. Results

### 3.1. Patients Summary

Table 1 shows the demographic data distribution for GS groups. The prospective data set comprised 200 patients clinically suspected of PCa. They were reclassified into three groups. Gleason score 3 + 3 were classified as Group 1; Gleason score 3 + 4 classified as Group 2; and 4 + 3 or 4 + 4 were classified as Group 3.

**Figure 1:**
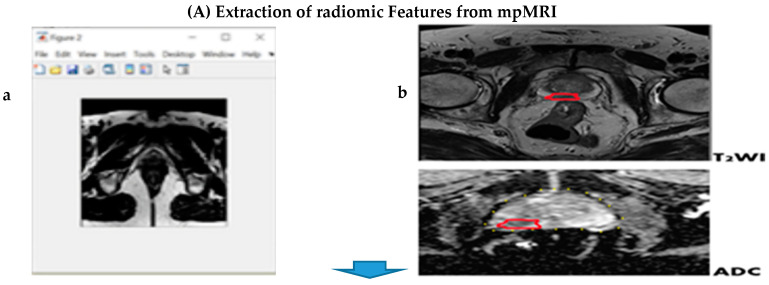

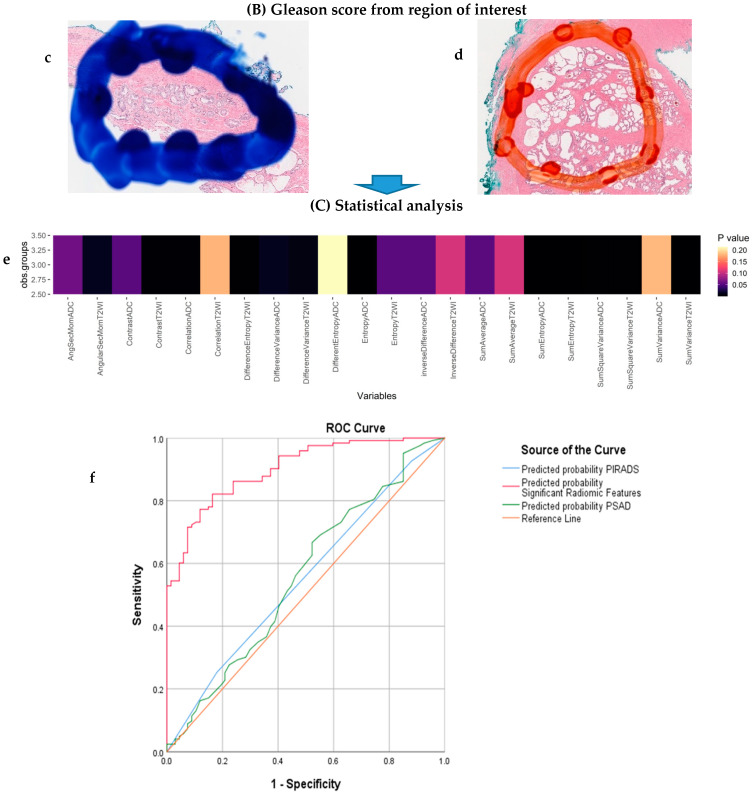
Research workflow. (**A**) mpMR images showing the segmented region of interest (ROI) marked red in both the T2WI and ADC images for extraction of quantitative imaging texture features (**a**,**b**). (**B**) Microscopic view of clinically significant prostate cancer on histological grading (Gleason’s score) (**c**,**d**). (**C**) Correlation showing significance analysis and AUC obtained by the linear regression models for predicting radiomic features with PIRADS: (**e**) Heatmap of the Kruskal–Wallis (after applying the Holm–Bonferroni correction) significant test *p*-values using radiomics features to identify patients of different GS. Significant features that were compared with the GS groups are shown in the colour black (corrected *p*-value < 0.05). (**f**) Receiver operating characteristics (ROC) curve and area under the curve (AUC) for model discriminative ability (the areas under the ROC are 0.551 for PIRADS, 0.901 for significant RF and 0.557 for PSAD).

**Figure 2:**
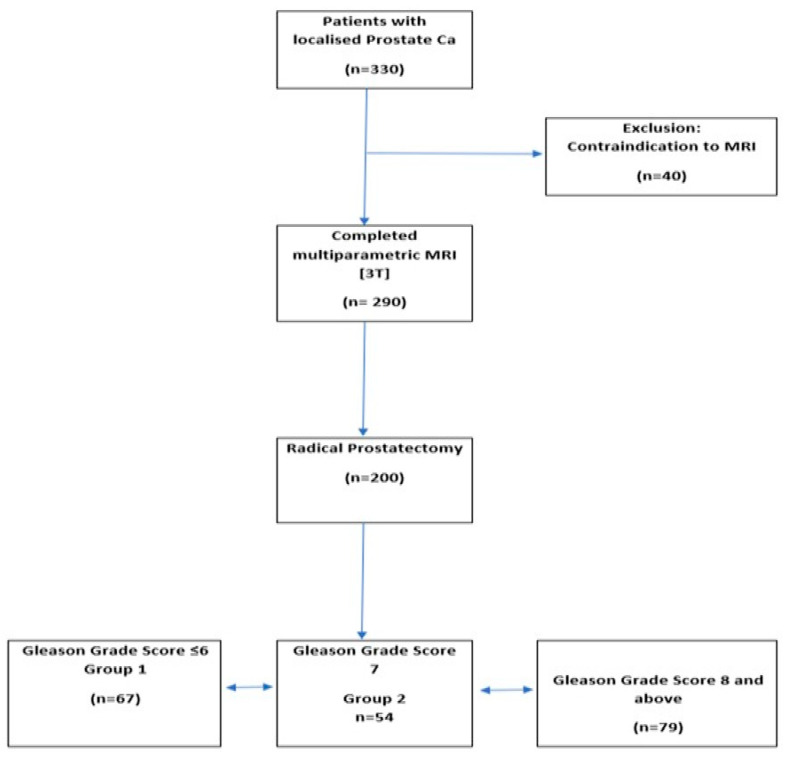
Study flowchat.

Figure 1 describes the research workflow and Figure 2 describes the study flowchart. A total of 200 patients who met the above-mentioned inclusion criteria were enrolled into this study.

### 3.2. Correlation Analysis

In Figure 1e, the Kruskal–Wallis test was applied, first to identify the significant radiomics features between the three groups of GS (i.e., G1, G2, and G3). Then, the Holm–Bonferroni method was applied to correct and control the probability of false rejections, by counteracting the problem of multiple comparisons in order to control the family-wise error rate. This permitted the discovery that 12 features out of the 22 radiomics features significantly correlated with the Gleason groups.

**Figure 3:**
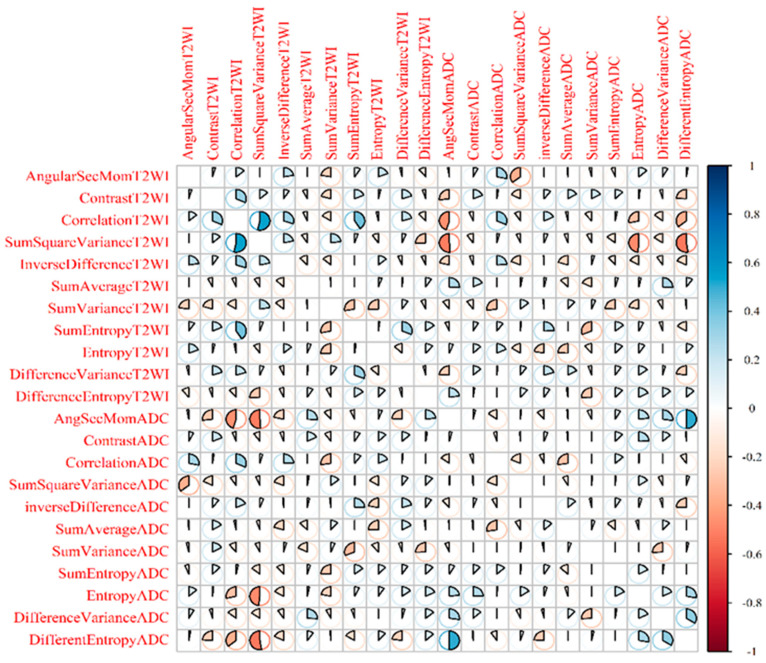
Spearman’s rank correlation between each of the radiomics features and the GS groups.

Figure 3 shows the Spearman’s rank correlation between each of the radiomics features and the GS groups. The values of the correlation were mostly between ±0.5 and ±0.5, indicating moderate correlation.

### 3.3. Significant Features

In univariate logistic regression, except for the Angular Second Moment T2WI and the Sum Square Variance ADC, all the other 10 radiomic features were significant predictors of clinically significant prostate cancer, as confirmed on radical prostatectomy (Table 2), and therefore, were put into multivariable analysis. The Sum Variance T2WI, Sum Entropy T2WI, Difference Variance T2WI, Entropy ADC and Difference Variance ADC were associated with clinically significant prostate cancer in the multiple logistic regression model. PSAD and PIRADS were tested in the univariate logistic regression stage, but the results were not statistically significant and therefore not included in the next stage.

### 3.4. Predictive Analysis

**Figure 4:**
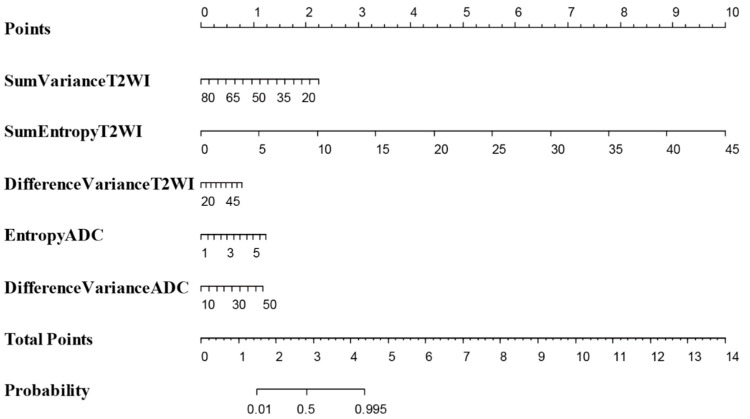
The statistically significant variables from the multivariable logistic regression model (Sum Variance T2WI, Sum Entropy T2WI, Difference Variance T2WI, Entropy ADC and Difference Variance ADC) were used to develop a nomogram to predict the probability of significant PCa.

The statistically significant variables from the multiple logistic regression model (Sum Variance T2WI, Sum Entropy T2WI, Difference Variance T2WI, Entropy ADC and Difference Variance ADC) were used to develop a nomogram to predict the probability of clinically significant prostate cancer (Figure 4).

The model demonstrated excellent discriminative ability (C-statistic = 0.901, 95%CI 0.859–0.943, Figure 1f). When comparing the probability of correctly predicting significant prostate cancer between explanatory radiomic features (Sum Variance T2WI, Sum Entropy T2WI, Difference Variance T2WI, Entropy ADC and Difference Variance ADC), PSAD and PIRADS via area under the ROC curve, radiomic features were 35.0% and 34.4% more successful than the PIRADS and PSAD, respectively, in correctly predicting significant prostate cancer in our patients (Table 3, *p* < 0.001).

**Figure 5:**
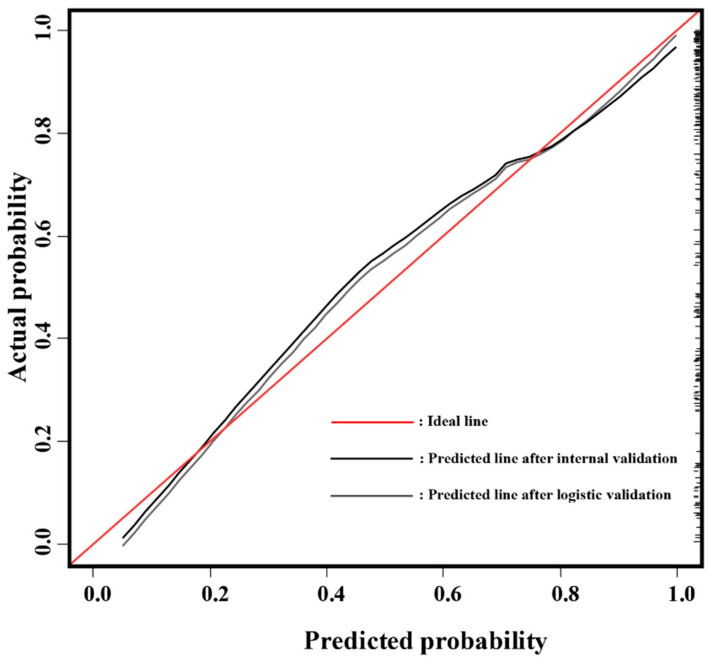
Calibration curves and internal validation of the nomogram (B = 200 boot repetitions, mean absolute error = 0.034. n = 200).

In addition to the AUROC, calibration analysis was applied to measure how far the predictions were from the actual outcomes. The calibration plot demonstrated good agreement between the model predictions and actual observations for detecting significant prostate cancer using statistically significant radiomic features, with only a limited departure from the ideal predictions. The mean absolute error was 3.4% when applying 200 times internal bootstrap correction. The results of the decision curve analysis are shown in Figure 5.

## 4. Discussion

This is the first prospective study to bring together information on radiomics features in pre-biopsy MRI and histopathological slides of RP specimens by utilizing the 3D-specific molds, thus bridging the gap in the existing literature. The primary outcome of the study was the diagnostic accuracy of the radiomics approach using GLCM texture features in predicting clinically significant prostate cancer. Our results show that when comparing the probability of correctly predicting significant prostate cancer between GLCM radiomic texture features (Sum Variance T2WI, Sum Entropy T2WI, Difference Variance T2WI, Entropy ADC and Difference Variance ADC), PSAD and PIRADS via area under the ROC curve, radiomic features were 35.0% and 34.4% more successful than PIRADS and PSAD, respectively, in correctly predicting the presence of clinically significant prostate cancer in our patient cohort (Table 3, *p* < 0.001). The nomogram in our model demonstrated an excellent discriminative ability (C-index 0.90). The use of mpMRI in evaluating PCa is attaining wider acceptance and our findings show that radiomics texture features extracted from radical prostatectomy can act as reliable quantitative imaging biomarkers for PCa detection and risk stratification [17,26]. The findings of the study become more interesting in the context where reports concerning the PIRADS scoring systems are conflicting, possibly due to the fact that the evaluation of MRI and scoring are operator dependent (detection biases due to subjectivity and inter-observer variability). In contrast, the use of radiomics features reduces such discrepancies as the features are automatically generated from the system output rather than via individual assessment. Our results are similar to observations reported in some retrospective studies [27,28,29]. We discovered that 12 out of the 22 radiomics features correlated with the Gleason groups, again a consistent finding with the previous studies. Two further studies using different methodologies to our study also reached similar conclusions and clearly delineate the potential value of radiomics in the prediction of GS [18,30]. When comparing the ability to correctly predict clinically significant PCa between radiomics features and PIRADS score, we did not include age and PSA in the model because both showed statistically non-significant results in the univariate analysis. The final model in the ROC curve comparison only focused on radiomics features (GLCM) versus PIRADS and PSAD in predicting significant PCa in our cohort (Table 3, *p* < 0.001). The Sum Entropy T2WI was demonstrated to have the greatest impact for predicting GS, as shown in Figure 4. Ultimately, Sum Entropy explains the degree of disorder or randomness of the texture within the PCa region, and the increase in values of Sum Entropy features are associated with the abnormality in texture due to the heterogeneity of the tumor region.

We observed that previous studies were retrospective analyses, while ours was a prospective study involving a pre-biopsy MRI focusing on the GLCM which measures the spatial relationship between neighboring voxels in predicting GS groups in clinically significant cancer [16,18,31,32,33,34]. Chaddad et al. [18] included T2-weighted (T2-WI) and apparent diffusion coefficient (ADC, computed from diffusion-weighted imaging) scans in their analysis, in a smaller number of 99 PCa patients. The cohort included an openly available imagery database. Based on the similar experience of previous studies, we focused on second-order texture features because they appeared to be the best feature for characterizing tumor heterogeneity [35].

Our study’s findings were consistent with another reported study [36], suggesting the radiomics score to have a higher significance in the area under the ROC curve when compared with the PIRADS system. However, we used RP specimens as a reference standard to eliminate bias which could be associated with the possible upgrading of the Gleason score between biopsy and RP specimen [37]. In their study, Slaoui et al. 2017 [32] correlated PIRADS v2 of mpMRI with the GS using RP specimens as a reference standard and found that the PIRADS system alone cannot predict GS in prostate cancer. This is similar to our findings at the univariate analysis stage. The other major difference between this study and our study, is that ours is a prospective study that used PIRADS score and radiomics with a better reference standard. Our results provide credence to the findings of the study by Algohary et al. [33], which evaluated the performance of radiomics features with clinically significant PCa of patients on active surveillance. Again, the corresponding study evidenced a limited cohort size of 56 patients, in addition to utilizing an MRI/TRUS fusion-guided biopsy as a reference standard—a contrast to our study.

The present study and a number of other groups have contributed to a body of evidence to suggest that quantitative imaging parameters using radiomics provide a better reflection of prostate cancer aggressivity than just visual inspection by clinicians. The missing piece of evidence, a prospective protocol-based study with the use of 3D-printed molds for a reference standard histology, has been added by the present study. This is a significant advancement which has the potential to be used in the risk stratification of prostate cancer, in particular, early localized disease where a number of options ranging from active monitoring to radical surgery exist. This research information should contribute to specialty-specific guidelines and wider implementation in the future.

Our study has some limitations. First, our analysis was performed using manually segmented ADC and T2WI MRI. Second, the tumors were not specified according to the zones of occurrence (transitional and peripheral zones) due to the small number of transitional zone lesions. Third, our mpMRI images were obtained from a single institution with experienced uro-radiologist readings. Further multicenter studies and external validation of models are required. In the future, we suggest using more image modalities, such as combining the radiomics model with gene expression in PCa, which could further improve the risk stratification of PCa. The addition of next-generation imaging (NGI) with Ga-PSMA PET/CT may improve our ability to predict risk at a higher level. Moreover, further machine learning in a larger dataset is required, with a view to generate automated systems for the diagnosis of clinically significant PCa in pre-biopsy mpMRI images.

## 5. Conclusions

This study concludes that GLCM texture features can be used to predict GS, with the Sum Entropy T2WI score having the greatest impact, followed by the Sum Variance T2WI. The findings support the hypothesis that radiomic analysis has the potential to be applied as a non-invasive marker for predicting GS and clinically significant PCa.

## Figures and Tables

**Table 1:** Demographic data and Gleason grouping.

Gleason Grade Score	Gleason Group	Number
Gleason Grade Score ≤6	Group 1	67
Gleason Grade Score 3 + 4 = 7	Group 2	54
Gleason Grade Score 4 + 3 = 7	Group 3	79
and above		

**Table 2:** Univariate and multivariable logistic regression analysis in predicting significant prostate cancer * (n = 200).

Covariate	Univariate Logistic Regression				Multivariable Logistic Regression			
	OR	95%CI		*p* Value	OR	95%CI		*p* Value
		Lower	Upper			Lower	Upper	
PSAD	6.889	0.500	94.847	0.149	-			
PI-RADS 3	Ref			0.356	-			
PI-RADS 4	1.570	0.568	4.342	0.385				
PI-RADS 5	2.296	0.718	7.342	0.161				
Angular Second Moment T2WI	1.529	0.219	10.678	0.668	-			
Contrast T2WI	1.023	1.007	1.040	0.005	1.017	0.993	1.041	0.168
Sum Square Variaqnce T2WI	0.976	0.965	0.988	<0.001	0.981	0.963	1.001	0.051
Sum Variance T2WI	0.905	0.877	0.933	<0.001	0.909	0.873	0.948	<0.001
Sum Entropy T2WI	1.923	1.417	2.609	<0.001	2.022	1.220	3.350	0.006
Difference Variance T2WI	1.056	1.024	1.090	0.001	1.068	1.015	1.124	0.011
Difference Entropy T2WI	1.278	1.020	1.601	0.033	1.065	0.776	1.463	0.696
Correlation ADC	8.400	1.998	35.308	0.004	5.030	0.766	33.050	0.093
Sum Square Variance ADC	1.002	0.986	1.018	0.839	-			
Sum Entropy ADC	1.504	1.095	2.066	0.012	1.103	0.702	1.732	0.672
Entropy ADC	2.667	1.691	4.208	<0.001	1.835	1.017	3.312	0.044
Difference Variance ADC	1.072	1.033	1.113	<0.001	1.105	1.042	1.172	0.001

* Significant prostate cancer was defined as prostate cancer with a Gleason Score ≥ 4 + 3.

**Table 3:** AUC comparison between radiomic features and PIRADS, and radiomic features and PSAD.

	Actual Significant PCa	Actual Non Significant PCa	AUC	Standard Error	Difference AUC	Standard Error of Difference	z Value	*p* Value
Radiomic Features	72	128	0.901	0.021	0.350	0.048	7.274	<0.001
PIRADS	67	123	0.551	0.044				
Radiomic Features	72	128	0.901	0.021	0.344	0.045	7.577	<0.001
PSAD	67	123	0.557	0.045				

## Data Availability

The data presented in this study are available on request from the corresponding author.

## Supplementary Materials

The following are available online at https://www.mdpi.com/article/10.3390/cancers13246199/s1, Table S1: Holm-Bonferroni adjustment following Kruskal-Wallis test. Table S2: Patients characteristics (n = 200). Table S3: GLCM T2WI texture features showing the Standard deviation, mean, number, median, minimum, and max-imum. Table S4: GLCM ADC texture features showing the Standard deviation, mean, number, median, minimum and maxi-mum. Table S5: Description of Gray-level co-occurrence Metrix (GLCM) features computed within the region of interest (ROI) of prostate tumour.

## References

[1] Bray F., Ferlay J., Soerjomataram I., Siegel R.L., Torre L.A., Jemal A. (2018). Global cancer statistics 2018: GLOBOCAN estimates of incidence and mortality worldwide for 36 cancers in 185 countries. CA Cancer J. Clin..

[2] Torre L.A., Bray F., Siegel R.L., Ferlay J., Lortet-Tieulent J., Jemal A. (2015). Global cancer statistics, 2012. CA Cancer J. Clin..

[3] Hoffman R.M., Gilliland F.D., Adams-Cameron M., Hunt W.C., Key C.R. (2002). Prostate-specific antigen testing accuracy in community practice. BMC Fam. Pract..

[4] Simpkin A.J., Rooshenas L., Wade J., Donovan J.L., Lane J.A., Martin R.M., Metcalfe C., Albertsen P.C., Hamdy F.C., Holmberg L. (2015). Development, Validation and Evaluation of an Instrument for Active Monitoring of Men with Clinically Localised Prostate Cancer: Systematic Review, Cohort Studies and Qualitative Study. Health Serv. Deliv. Res..

[5] Welch H.G., Schwartz L.M., Woloshin S. (2005). Prostate-specific antigen levels in the United States: Implications of various definitions for abnormal. J. Natl. Cancer Inst..

[6] Narain V., Bianco F.J., Grignon D.J., Sakr W.A., Pontes J.E., Wood D.P. (2001). How accurately does prostate biopsy Gleason score predict pathologic findings and disease free survival?. Prostate.

[7] Melia J., Moseley R., Ball R.Y., Griffiths D.F.R., Grigor K., Harnden P., Parkinson M.C. (2006). A UK-based investigation of inter-and intra-observer reproducibility of Gleason grading of prostatic biopsies. Histopathology.

[8] Short E., Warren A.Y., Varma M. (2019). Gleason grading of prostate cancer: A pragmatic approach. Diagn. Histopathol..

[9] Hu J.C., Gu X., Lipsitz S.R., Barry M.J., D’Amico A.V., Weinberg A.C., Keating N.L. (2009). Comparative effectiveness of minimally invasive vs open radical prostatectomy. JAMA.

[10] Bjurlin M.A., Wysock J.S., Taneja S.S. (2014). Optimization of prostate biopsy: Review of technique and complications. Urol. Clin..

[11] Lojanapiwat B., Anutrakulchai W., Chongruksut W., Udomphot C. (2014). Correlation and diagnostic performance of the prostate-specific antigen level with the diagnosis, aggressiveness, and bone metastasis of prostate cancer in clinical practice. Prostate Int..

[12] Liu B., Cheng J., Guo D., He X., Luo Y., Zeng Y., Li C. (2019). Prediction of prostate cancer aggressiveness with a combination of radiomics and machine learning-based analysis of dynamic contrast-enhanced MRI. Clin. Radiol..

[13] Weinreb J.C., Barentsz J.O., Choyke P.L., Cornud F., Haider M.A., Macura K.J., A Margolis D.J., Schnall M.D., Shtern F., Tempany C.M. (2016). PI-RADS Prostate Imaging–Reporting and Data System: 2015, Version 2. Eur. Urol..

[14] Mehralivand S., Shih J.H., Rais-Bahrami S., Oto A., Bednarova S., Nix J.W., Thomas J.V., Gordetsky J.B., Gaur S., Harmon S.A. (2018). A Magnetic Resonance Imaging–Based Prediction Model for Prostate Biopsy Risk Stratification. JAMA Oncol..

[15] Donati O.F., Afaq A., Vargas H.A., Mazaheri Y., Zheng J., Moskowitz C.S., Hricak H., Akin O. (2014). Prostate MRI: Evaluating tumor volume and apparent diffusion coefficient as surrogate biomarkers for predicting tumor Gleason score. Clin. Cancer Res..

[16] Fehr D., Veeraraghavan H., Wibmer A., Gondo T., Matsumoto K., Vargas H.A., Sala E., Hricak H., Deasy J.O. (2015). Automatic classification of prostate cancer Gleason scores from multiparametric magnetic resonance images. Proc. Natl. Acad. Sci. USA.

[17] Alexandratou E., Yova D., Gorpas D., Maragos P., Agrogiannis G., Kavantzas N. (2008). Texture analysis of tissues in Gleason grading of prostate cancer. Imaging, Manipulation, and Analysis of Biomolecules, Cells, and Tissues VI.

[18] Chaddad A., Niazi T., Probst S., Bladou F., Anidjar M., Bahoric B. (2018). Predicting Gleason Score of Prostate Cancer Patients Using Radiomic Analysis. Front. Oncol..

[19] Nketiah G., Elschot M., Kim E., Teruel J.R., Scheenen T.W., Bathen T.F., Selnæs K.M. (2017). T2-weighted MRI-derived textural features reflect prostate cancer aggressiveness: Preliminary results. Eur. Radiol..

[20] Ploussard G., Epstein J.I., Montironi R., Carroll P.R., Wirth M., Grimm M.O., Bjartell A.S., Montorsi F., Freedland S.J., Erbersdobler A. (2011). The contemporary concept of significant versus insignificant prostate cancer. Eur. Urol..

[21] Alqahtani S., Wei C., Zhang Y., Szewczyk-Bieda M., Wilson J., Huang Z., Nabi G. (2020). Prediction of prostate cancer Gleason score upgrading from biopsy to radical prostatectomy using pre-biopsy multiparametric MRI PIRADS scoring system. Sci. Rep..

[22] Sheikh N., Wei C., Szewczyk-Bieda M., Campbell A., Memon S., Lang S., Nabi G. (2017). Combined T2 and diffusion-weighted MR imaging with template prostate biopsies in men suspected with prostate cancer but negative transrectal ultrasound-guided biopsies. World J. Urol..

[23] Wei C., Jin B., Szewczyk-Bieda M., Gandy S., Lang S., Zhang Y., Huang Z., Nabi G. (2018). Quantitative parameters in dynamic contrast-enhanced magnetic resonance imaging for the detection and characterization of prostate cancer. Oncotarget.

[24] Epstein J.I., Allsbrook W.C., Amin M.B., Egevad L.L., ISUP Grading Committee (2005). The 2005 International Society of Urological Pathology (ISUP) consensus conference on Gleason grading of prostatic carcinoma. Am. J. Surg. Pathol..

[25] Epstein J.I., Egevad L., Amin M.B., Delahunt B., Srigley J.R., Humphrey P.A. (2016). The 2014 International Society of Urological Pathology (ISUP) consensus conference on Gleason grading of prostatic carcinoma. Am. J. Surg. Pathol..

[26] Nketiah G.A., Elschot M., Scheenen T.W., Maas M.C., Bathen T.F., Selnæs K.M. (2021). Utility of T 2-weighted MRI texture analysis in assessment of peripheral zone prostate cancer aggressiveness: A single-arm, multicenter study. Sci. Rep..

[27] Chen T., Li M., Gu Y., Zhang Y., Yang S., Wei C., Wu J., Li X., Zhao W., Shen J. (2019). Prostate cancer differentiation and aggressiveness: Assessment with a radiomic-based model vs. PI-RADS v2. J. Magn. Reson. Imaging.

[28] Barucci A., Bastiani P., Carpi R., Fondelli S., Giannetti A., Olmastroni M., Pini R., Ratto F., Rucco M., Zatelli G. (2018). Prostate cancer Radiomics using multiparametric MR imaging: An exploratory study. Phys. Medica Eur. J. Med. Phys..

[29] Woźnicki P., Westhoff N., Huber T., Riffel P., Froelich M.F., Gresser E., von Hardenberg J., Mühlberg A., Michel M.S., Schoenberg S.O. (2020). Multiparametric MRI for prostate cancer characterization: Combined use of radiomics model with PI-RADS and clinical parameters. Cancers.

[30] Hectors S.J., Cherny M., Yadav K.K., Beksaç A.T., Thulasidass H., Lewis S., Davicioni E., Wang P., Tewari A.K., Taouli B. (2019). Radiomics features measured with multiparametric magnetic resonance imaging predict prostate cancer aggressiveness. J. Urol..

[31] Haralick R.M., Shanmugam K., Dinstein I. (1973). Textural Features for Image Classification. IEEE Trans. Syst. Man Cybern..

[32] Slaoui H., Neuzillet Y., Ghoneim T., Rouanne M., Abdou A., Lugagne-Delpon P.M., Scherrer A., Radulescu C., Delancourt C., Molinie V. (2017). Gleason score within prostate abnormal areas defined by multiparametric magnetic resonance imaging did not vary according to the PIRADS score. Urol. Int..

[33] Algohary A., Viswanath S., Shiradkar R., Ghose S., Pahwa S., Moses D., Jambor I., Shnier R., Böhm M., Haynes A.M. (2018). Radiomic features on MRI enable risk categorization of prostate cancer patients on active surveillance: Preliminary findings. J. Magn. Reson. Imaging.

[34] Li J., Weng Z., Xu H., Zhang Z., Miao H., Chen W., Liu Z., Zhang X., Wang M., Xu X. (2018). Support Vector Machines (SVM) classification of prostate cancer Gleason score in central gland using multiparametric magnetic resonance images: A cross-validated study. Eur. J. Radiol..

[35] Chitalia R.D., Kontos D. (2019). Role of texture analysis in breast MRI as a cancer biomarker: A review. J. Magn. Reson. Imaging.

[36] Li M., Yang L., Yue Y., Xu J., Huang C., Song B. (2021). Use of Radiomics to Improve Diagnostic Performance of PI-RADS v2.1 in Prostate Cancer. Front. Oncol..

[37] Chun F., Steuber T., Erbersdobler A., Currlin E., Walz J., Schlomm T., Haese A., Heinzer H., McCormack M., Huland H. (2006). Development and Internal Validation of a Nomogram Predicting the Probability of Prostate Cancer Gleason Sum Upgrading Between Biopsy and Radical Prostatectomy Pathology. Eur. Urol..

