# Supplementary material for: Prediction of Clinically Significant Cancer Using Radiomics Features of Pre-Biopsy of Multiparametric MRI in Men Suspected of Prostate Cancer
Source: Cancers (Basel). 2021 Dec 9;13(24):6199. doi: 10.3390/cancers13246199 (PMC8699138; doi:10.3390/cancers13246199)
Supplement: Supplementary file 1 [file cancers-13-06199-s001.zip › cancers-1377337-supplementary.pdf]

cancers

MDPI

Supplementary Materials: Prediction of Clinically Significant Cancer Using Radiomics Features of Pre-Biopsy of Multiparametric MRI in Men Suspected of Prostate Cancer

Chidozie N. Ogbonnaya, Xinyu Zhang, Basim S. O. Alsaedi, Norman Pratt, Yilong Zhang, Lisa Johnston and Ghulam Nabi

Table S1. Holm-Bonferroni adjustment following Kruskal-Wallis test.

Obs.tot	Obs group	df	statistic	p-value	holmBonf	Variance
200	3	2	77.05261	1.855E-17	4.08E-16	SumVarianceT2WI
200	3	2	40.29552	1.778E-09	3.734E-08	SumEntropyT2WI
200	3	2	30.26405	2.681E-07	5.361E-06	EntropyADC
200	3	2	22.04339	1.634E-05	0.000311	SumEntropyADC
200	3	2	20.48376	3.565E-05	0.000642	DifferenceEntropyT2WI
200	3	2	19.79669	5.026E-05	0.000854	SumSquareVarianceT2WI
200	3	2	18.80068	8.27E-05	0.001323	SumSquareVarianceADC
200	3	2	16.69491	0.000237	0.003555	CorrelationADC
200	3	2	16.5606	0.000253	0.003555	ContrastT2WI
200	3	2	14.46591	0.000722	0.009391	DifferenceVarianceT2WI
200	3	2	13.56251	0.001135	0.013618	AngularSecMomT2WI
200	3	2	13.53896	0.001148	0.013618	DifferenceVarianceADC
200	3	2	10.48425	0.005289	0.05289	ContrastADC
200	3	2	10.31543	0.005755	0.05289	SumAverageADC
200	3	2	10.26487	0.005902	0.05289	EntropyT2WI
200	3	2	10.22021	0.006035	0.05289	InverseDifferenceADC
200	3	2	9.150617	0.010303	0.061819	AngSecMomADC
200	3	2	7.67837	0.021511	0.107556	SumAverageT2WI
200	3	2	7.555213	0.022877	0.107556	InverseDifferenceT2WI
200	3	2	5.623613	0.060096	0.180289	CorrelationT2WI
200	3	2	4.792758	0.091047	0.182094	SumVarianceADC
200	3	2	3.085075	0.213838	0.213838	DifferentEntropyADC

Table S2: Patients characteristics (n=200).

	S/N	AGE	PSA	Volume	PSAD	Significant Gleason score	PIRADS
Stand dev	57.86312	6.262884	4.984889	29.19453	0.115035	0.481205	0.666583
Mean	100.49	63.82	11.085	66.84475	0.19	0.64	4.589474
n	200	200	200	200	200	200	200
Median	100.5	64	10.25	59.75	0.16	1	5
Minimum	1	45	1.1	20	0.01	0	2
Maximum	200	75	21	207	0.56	1	5

Table S3. GLCM T2WI texture features showing the Standard deviation, mean, number, median, minimum, and maximum.

AngularSecMomT2WI	ContrastT2WI	CorrelationT2WI	SumSquareVarianceT2WI	InverseDifferenceT2WI	SumAverageT2WI	SumVarianceT2WI	SumEntropyT2WI	EntropyT2WI	DifferenceVarianceT2WI	DifferenceEntropyT2WI
0.150208	25.60642	0.181285	27.0797	0.179048	12.7754	13.19336	4.176457	1.149206	9.765953	1.348403
0.4005	52.36	0.5505	73.42	0.3235	67.015	41.975	3.8455	2.623	40.445	3.051
200	200	200	200	200	200	200	200	200	200	200
0.4	50	0.6	75.5	0.2	65	40.5	3.5	2.4	41	3.1
0.1	15	0.2	20	0.1	20	10	1.1	1	20	1.1
0.9	188	0.9	146	0.9	99	80	44	6.1	57	7.2

Table S4. GLCM ADC texture features showing the Standard deviation, mean, number, median, minimum and maximum.

AngSecMomADC	ContrastADC	CorrelationADC	SumSquareVarianceADC	inverseDifferenceADC	SumAverageADC	SumVarianceADC	SumEntropyADC	EntropyADC	DifferenceVarianceADC	DifferentEntropyADC
0.739036	18.50003	0.222127	17.92867	0.191422	20.40981	16.66998	0.964133	1.039752	9.070676	1.166338
0.7575	50.49	0.4675	74.745	0.329	39.55	41.785	2.686	1.961	29.435	2.0825
200	200	200	200	200	200	200	200	200	200	200
0.4	47	0.4	80	0.3	36.5	40	2.6	1.4	27	1.5
0.1	18	0.1	40	0.1	6	10	1	1	10	1
3.9	94	0.9	107	0.9	93	93	5	5.6	50	5

Table S5. Description of Gray-level co-occurrence Metrix (GLCM) features computed within the region of interest (ROI) of prostate tumour.

Texture Features (2nd order statistic Characterizing spatial relationship between intensity values within ROIs).	Description
Angular Second moment	Measures the textural uniformity that is pixel pair recurrences. It detects disorder in textures
Contrast	Measures local intensity variations
Correlation	Explains the linear dependency of Grey level values to their respective voxels in the GLCM
Sum Square Variance	Measures the distribution of neighbouring intensity level pairs about the average of intensity level in the GLCM
Inverse Difference	Measures of the local homogeneity of an image
Sum Average	Measures the relationship between occurrences of pairs with lower intensity values and occurrences of pairs with higher intensity values
Sum Variance	Explains the weights elements that differ from the average value of the GLCM.
Sum Entropy	Explains the sum of neighbourhood intensity value differences.
Entropy	Explains the randomness in the GLCM
Difference Variance	Measure of heterogeneity that places higher weights on differing intensity level pairs that deviate more from the mean.
Difference Entropy	Measure of the randomness/variability in neighbourhood intensity value differences.
